# 

**Published:** 1852-03

**Authors:** 


### CONTENTS

OF THE

### MEDICAL EXAMINER.

NEW SERIES—NO. LXXXVII.—MARCH, 1852.

### ORIGINAL COMMUNICATIONS.

On the Organizing of the American Medical Association. Read before the Philadelphia County Medical Society, Feb., 1852. By the President, Samuel Jackson, M. D., formerly of Northumberland. Printed by order of the Society, - - 137

Fractures of the lower end of the Radius, and on their Management. By H. Bond, M. D., Philadelphia. (Read before the Philadelphia College of Physicians and published in their "Transactions,") - - - - - - - 146

Case of Fracture of the Anterior Inferior Spinous Process of the Ilium. By Charles W. Ashby, M. D., of Culpepper Co., Va. (Communicated by Professor Mütter,) - - - 159

### CLINICAL REPORTS.

Clinic of the Jefferson Medical College. Service of Professor Dunglison, - - - - - - - 162

### BIBLIOGRAPHICAL NOTICES.

Diseases of the Skin elucidated by Anatomical Investigations. By Dr. Gustavus Simon, Directing Physician to the Charity Hospital, and privatim docens in the University of Berlin, - 170

Manual of Diseases of the Skin, from the French of MM. Cazenave and Schedel. With Notes and Additions, by Thomas H. Burgess, M. D., Surgeon to the Blenheim street Dispensary for Diseases of the Skin, &c. Second American edition, enlarged and corrected from the last French edition, with additional Notes, by H. D. Bulkley, M. D., Physician to the New York Hospital; Fellow of the College of Physicians and Surgeons, New York; Lecturer on Diseases of the Skin, &c. &c., - 170

Report of the Pennsylvania Hospital for the Insane, for the year 1851. By Thomas S. Kirkbride, M. D., Physician to the Institution, 177

Remarks and Recommendations on the Professional Education of Dentists. By John Trenor, M. D., of New York. [From the New York Journal of Medicine,] - - - - 180

Essays on Life, Sleep and Pain, etc. By Samuel Henry Dickson, M. D., Professor of Institutes and Practice of Medicine in the Medical College of the State of South Carolina, - - 180

A Treatise on the Diseases of the Chest, being a Course of Lectures delivered at the New York Hospital. By John A. Swett, M. D., Physician to the New York Hospital, - - - - 181

### EDITORIAL.

Charleston Preparatory Medical School, - - - - - 181

Election of Officers of the Northern Medical Association, - - 183

Circular of the American Medical Society in Paris, - - - 184

### MEDICAL NEWS.

Pennsylvania Hospital, - - - - - - - 184

Medical Department of the Army, - - - - - 184

Board of Naval Surgeons, - - - - - - - 185

Dr. Valentine Mott, and the Professorship of Surgery, - - 185

Extramural Interments in London, - - - - - - 185

The Mummy found in St. Stephen's Crypt, Westminster, - - 185

Death of M. Gannal, - - - - - - - 186
Naval Assistant Surgeons, - - - - - - - 186
Mr. Syme's Originality, - - - - - - - 187
A Brazen Homœopath, - - - - - - - 188
Medical Charities, - - - - - - - - 188
Honors of Medical Men, - - - - - - - 189
Munificent Donation, - - - - - - - 189
Death of Baron Pasquier, - - - - - - - 188
Medical Museums, - - - - - - - - 189
International Sanitary Convention in Paris, - - - - - 190
Arsenic Eaters, - - - - - - - - 190

### RECORD OF MEDICAL SCIENCE.

### SURGERY.

Case of a Foreign Body in the Air-Passages; Tracheotomy performed for its removal. Expulsion of the body twenty-eight days after the Operation, and recovery of the Patient. By David Johnston, A. M., Surgeon to the Royal Infirmary, Montrose, - 192
Removal of a Nævus by a Platinum Wire, heated by a Galvanic Current, - - - - - - - - 197
A Substitute for Mercury in Syphilitic Diseases, - - - 198
Dr. Lebert on the Structure of Cataract, - - - - - 198

### PATHOLOGY AND PRACTICE OF MEDICINE.

Hereditary Insanity, - - - - - - - - 199
Poisoning by Datura Stramonium, - - - - - - 199
Causes of Albuminous Urine, - - - - - - - 201
Strangulated Hernia reduced during Vomiting, - - - - 202

### ANATOMY AND PHYSIOLOGY.

Why is a true Corpus Luteum formed only in cases of Impregnation, 203
On the Employment of Sulphate of Zinc as an antiseptic, - - 203
Monstrosity, - - - - - - - - - 203
On the transportation of the two Aortæ into one in Embryonic Vertebrata, - - - - - - - - 204

### MATERIA MEDICA AND THERAPEUTICS.

Tartaric Acid used for aiding the Solution of Disulphate of Quinine, 204

